# Somato‐Cognitive Action Network in Focal Dystonia

**DOI:** 10.1002/mds.70021

**Published:** 2025-08-28

**Authors:** Yuchao Wang, Baothy Huynh, Jianxun Ren, Mo Chen, Wei Zhang, Dan Hu, Shasha Li, Hesheng Liu, Teresa J. Kimberley

**Affiliations:** ^1^ Center for Cognitive Neuroscience Duke University Durham North Carolina USA; ^2^ Department of Physical Therapy MGH Institute of Health Professions Boston Massachusetts USA; ^3^ Department of Rehabilitation Science MGH Institute of Health Professions Boston Massachusetts USA; ^4^ Changping Laboratory Beijing China; ^5^ Neurosciences Research Program, Gillette Children's Specialty Healthcare St Paul Minnesota USA; ^6^ Non‐invasive Neuromodulation Laboratory University of Minnesota Minneapolis Minnesota USA; ^7^ Academy for Advanced Interdisciplinary Studies Peking University Beijing China; ^8^ Department of Radiology and Biomedical Research Imaging Center University of North Carolina at Chapel Hill Chapel Hill North Carolina USA; ^9^ Athinoula A. Martinos Center for Biomedical Imaging, Department of Radiology Massachusetts General Hospital Boston Massachusetts USA; ^10^ Department of Radiology Harvard Medical School Boston Massachusetts USA; ^11^ Biomedical Pioneering Innovation Center Peking University Beijing China

**Keywords:** task‐specific dystonia, fMRI, connectivity, precision functional mapping, disinhibition, motor cortex, cerebellum

## Abstract

**Background:**

The central pathology causing idiopathic focal dystonia remains unclear. The recently identified somato‐cognitive action network (SCAN) has been implicated.

**Objective:**

We tested whether the effector‐agnostic SCAN may constitute a central pathology shared across dystonia subtypes, whereas the effector‐specific regions in the primary sensorimotor cortex may show distinct functional changes specific to the dystonic body part.

**Methods:**

We collected functional magnetic resonance imaging (MRI) from patients with focal dystonia (laryngeal dystonia [LD], N = 24; focal hand dystonia [FHD], N = 18) and healthy control participants (N = 21). Regions of interest were selected a priori within the basal ganglia‐thalamo‐cortical and cerebello‐thalamo‐cortical sensorimotor pathways. We investigated dystonia‐dependent resting‐state connectivity changes: between SCAN and related cortical regions, between cortical and noncortical regions, and among noncortical regions. Cortical network boundaries were individualized based on resting‐state data. Separately, individualized hand and mouth/larynx regions were also generated from task‐based MRI (finger‐tapping and phonation, respectively) for comparison.

**Results:**

Both focal dystonia subtypes showed significant functional changes (*P* = 0.048 for LD, *P* = 0.017 for FHD) compared to controls, driven by SCAN's higher functional connectivity to task‐based mouth/larynx region and concomitantly lower connectivity to the cingulo‐opercular network. No significant subcortical or cerebellar changes were observed when LD and FHD were modeled as independent groups. However, exploratory analysis combining LD and FHD suggested a dystonia‐dependent asynchronization between SCAN and sensorimotor cerebellum (*P* = 0.010) that may indicate a pathological rather than compensatory process.

**Conclusions:**

We demonstrate that SCAN is uniquely associated with focal dystonia dysfunction beyond the dystonic effector regions, offering insights into pathophysiology and treatments. © 2025 The Author(s). *Movement Disorders* published by Wiley Periodicals LLC on behalf of International Parkinson and Movement Disorder Society.

A somato‐cognitive action network (SCAN) involving the primary motor cortex was recently revealed through individualized functional parcellation.^1^ This network, which interdigitates between the effector‐specific regions of hand, foot, and mouth in the motor cortex, is hypothesized to play a key role in movement planning and coordination, which may be disrupted in various movement disorders. Functionally, SCAN is widely engaged in movements across muscle groups, but is particularly activated during evolutionarily early, gross motor actions (eg, swallowing) that may be crucial preparatory steps for fine motor control (eg, speaking). SCAN also mediates between the goal‐directed cingulo‐opercular network (CON) and motoneuron‐directed effector‐specific regions,^2^ establishing a novel mechanism from cognitive intention to motor execution. Owing to its broad theoretical scope, SCAN has attracted growing interest in movement disorder research,^3^, ^4^ though whether and how it maps onto distinct disease features remains unclear.

Humans can experience discoordination and localized, abnormal muscle contractions (eg, in their laryngeal or hand muscles) that disrupt the ability to speak, play an instrument, or write, in the absence of neurological insult or other symptoms. This disease, known as idiopathic focal dystonia, has common subtypes such as laryngeal dystonia (LD) and focal hand dystonia (FHD).^5^ Focal dystonia is increasingly recognized as a network disorder involving sensorimotor and possibly cognitive‐affective functions, rather than solely a motor disease.^6^, ^7^, ^8^, ^9^, ^10^, ^11^ Still, most neuroimaging studies identify dystonia abnormalities along the basal ganglia‐thalamo‐cortical and cerebello‐thalamo‐cortical pathways (Fig. 1A).^5^, ^12^, ^13^, ^14^, ^15^, ^16^, ^17^, ^18^ Given SCAN's similar connectivity profile (the motor cortex to putamen, centromedian thalamus, and sensorimotor cerebellum) and its proposed role in dystonia‐related tasks (eg, speech and writing),^1^ there is a strong rationale to examine its integration in these circuits. Yet, SCAN's role has not been directly tested in focal dystonia.

**FIG. 1:**
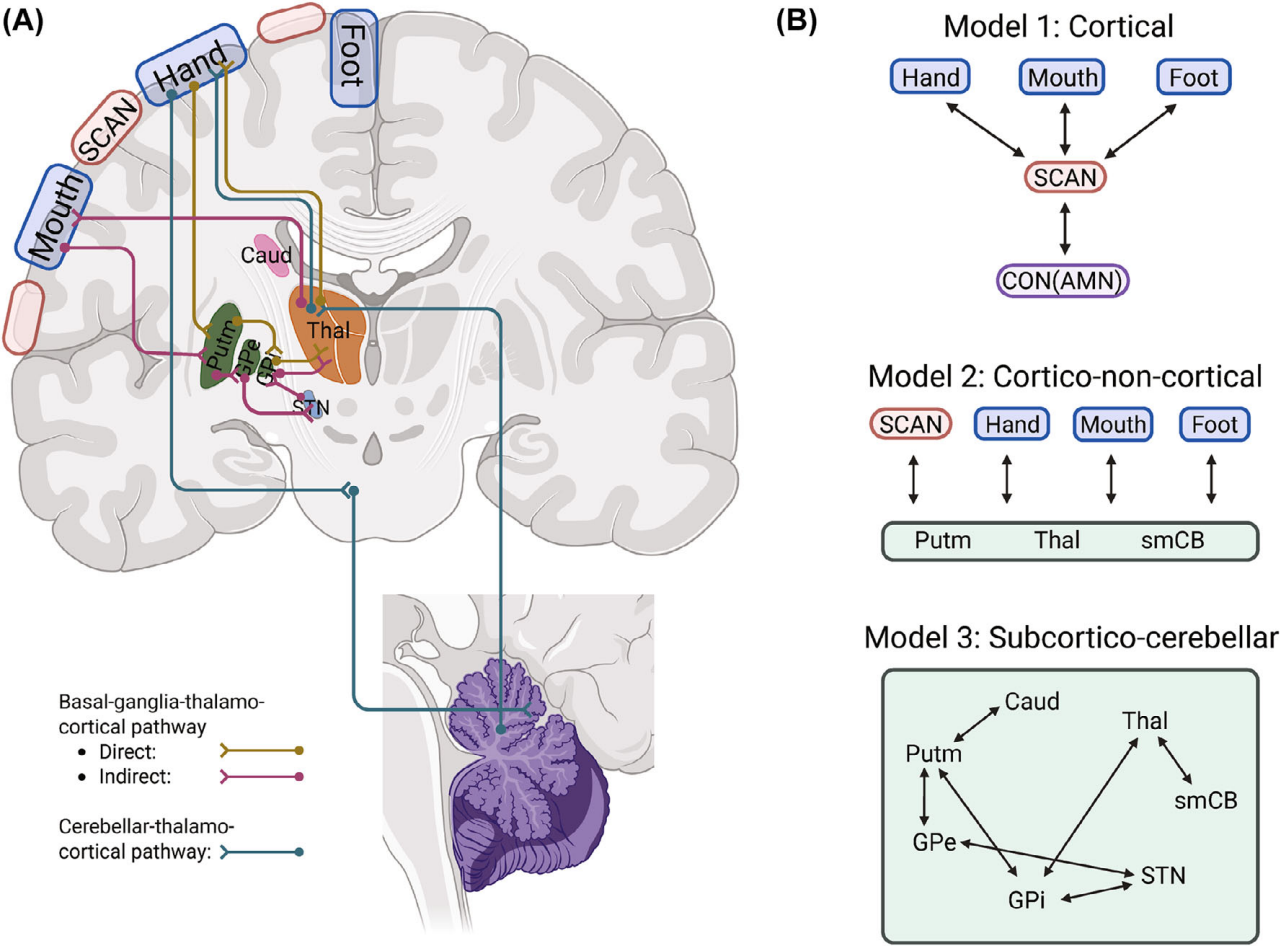
Overview of cortical–subcortical‐cerebellar pathways in sensorimotor control. The recently revealed somato‐cognitive action network (SCAN) may be involved in focal dystonia pathophysiology. (**A**) Simplified basal ganglia‐thalamo‐cortical and cerebello‐thalamo‐cortical pathways are shown with the relevant subcortical structures and cerebellum. The effector‐specific (hand, foot, mouth) regions are interdigitated by the SCAN in the primary motor cortex (bilaterally, with only the left hemisphere labeled). (**B**) Hypothesized functional connectivity (in black arrows) that may demonstrate different SCAN versus effector region involvement in focal dystonia affecting different body parts. We also explored connectivity within subcortical‐cerebellar circuitry. AMN, action‐mode network; Caud, caudate; CON, cingulo‐opercular network; FC, functional connectivity; GPe, globus pallidus externus; GPi, globus pallidus internus; Putm, putamen; smCB, sensorimotor cerebellum; STN, subthalamic nucleus; Thal, thalamus. Created in BioRender. Wang, Y. (2025) https://BioRender.com/z11g018. [Color figure can be viewed at wileyonlinelibrary.com]

Emergent evidence implicates SCAN in both the etiology and treatment of dystonia. In a pediatric population with lesion‐induced dystonia, symptom‐inducing lesions (in the putamen and globus pallidus) shared functional connectivity to SCAN.^19^ In another study investigating where subthalamic deep brain stimulation (DBS) targets were most effective among idiopathic dystonia subtypes, greater symptom improvement for axial dystonia (affecting the mouth, neck, or trunk) was associated with positive functional connectivity with CON,^20^ which is closely connected to SCAN. In contrast, CON connectivity was not observed for appendicular (arm and leg) dystonia, whose DBS targets mapped to the effector‐specific regions. It was posited that CON‐SCAN involvement in dystonia may differ between axial and appendicular symptoms,^20^ a distinction that warrants further study.

Broadly, it remains unclear whether subtypes of focal dystonia (eg, LD and FHD) share similar central pathology. Despite theories that the disrupted sensory feedback between the thalamus and primary somatosensory cortex is a key etiological factor,^8^, ^21^, ^22^, ^23^ no empirical work to our knowledge has compared the functional sensorimotor circuitry across dystonia subtypes. Among the limited studies that included multiple dystonia subtypes, most focused on a single brain structure (eg, the striatum,^24^ dentate nucleus^25^) or conducted whole‐brain analyses,^26^, ^27^, ^28^, ^29^, ^30^ making it challenging to isolate effector regions and disentangle network‐level changes. To address these challenges, we defined regions of interest (ROI) that distinguished dystonic and nondystonic body parts, and systematically tested cortical, subcortical, and cerebellar functional interactions in a hypothesis‐driven manner.

With a novel dataset comprising patients with LD or FHD and healthy controls in the current study, we tested the following hypotheses: (1) SCAN is associated with focal dystonia pathology, and (2) SCAN is more relevant for LD (axial) rather than FHD (appendicular focal dystonia).^20^ We also hypothesized that (3) focal dystonia dysfunction can be localized in sensorimotor control pathways in a dystonic‐body‐part‐specific manner. We used functional MRI (fMRI), which has demonstrated cortico‐subcortical‐cerebellar functional connectivity following neuroanatomy.^31^, ^32^, ^33^, ^34^ Given that LD and FHD symptoms occur only during tasks (ie, task specificity), and that functional network boundaries may differ between subjects and change during task performance,^14^, ^35^, ^36^, ^37^, ^38^, ^39^ we generated individualized effector ROIs for hand and mouth (including the larynx) from both resting‐state and task‐based fMRI data (finger‐tapping and phonation) independently for comparison. Individualized foot ROI from resting‐state data was included in the statistical model as a nonaffected control. We included atlas‐based subcortical and cerebellar regions to investigate dystonia‐related functional changes (Fig. 1B).

## Patients and Methods

### Participants

Sixty‐three participants were enrolled in the current study, including 24 people with LD (age = 60.5 ± 11.1 years, 17 females), 18 subjects with FHD (age = 55.1 ± 14.6 years, 6 females), and 21 healthy controls (age = 53.4 ± 12.7 years, 5 females) (Table 1). The study was approved by the University of Minnesota and Mass General Brigham Institutional Review Boards, and all participants provided written informed consent according to the Declaration of Helsinki. All subjects with focal dystonia were at least 3 months away from their last botulinum toxin injection and were symptomatic during their affected tasks. Two subjects (both from the LD group) were removed from subsequent analysis due to excessive motion (mean relative motion >0.4 mm during resting‐state scans). Dystonia symptom severity was measured with self‐report on the Voice Handicap Index (VHI)^40^ for LD and the Arm Dystonia Disability Scale (ADDS)^41^ for FHD.

**TABLE 1:** Overview of subject groups

	Laryngeal dystonia (N = 24)	Focal hand dystonia (N = 18)	Control (N = 21)	*P*‐Value
Sex	Female: 17, male: 7	Female: 6, male: 12	Female: 5, male: 16	LD vs. FHD: <0.05 LD vs. control: <0.01 FHD vs. control: 0.76
Age (years)	60.5 ± 11.1	55.1 ± 14.6	53.4 ± 12.7	≥0.06
Symptom duration (years)	17.2 ± 10.1	18.7 ± 15.3	N/A	0.83
Symptom type	Abductor: 4, adductor: 20	Left hand only: 6, right hand only: 9, bilateral hands: 3	N/A	N/A
Resting‐state scan mean relative motion (mm)	0.15 ± 0.10	0.14 ± 0.06	0.12 ± 0.07	0.52
Severity	Voice handicap index: 61.8 ± 18.1	Arm dystonia disability scale (%): 63.1 ± 17.2	N/A	N/A

*Note*: Measurements are presented as mean ± standard deviation (SD).

Abbreviations: LD, laryngeal dystonia; FHD, focal hand dystonia.

### MRI Acquisition

Structural and functional MRI was collected with a 3 T Siemens Magnetom Prisma fit scanner with a 32‐channel head coil (Siemens Healthineers, Forchheim, Germany). T_1_‐weighted structural imaging used a three‐dimensional (3D) multi‐echo magnetization‐prepared rapid gradient‐echo imaging (MPRAGE) sequence (repetition time (TR) = 2.5 seconds, echo time (TE) = 1.81, 3.6, 5.39, 7.18 ms, inversion time = 1.0 seconds, 0.8‐mm isotropic voxels, field of view (FoV) = 256 mm, 208 sagittal slices, flip angle = 8°, bandwidth = 740 Hz/Px). Hand and vocal task‐based fMRI were obtained using multiband gradient‐echo echo‐planar imaging (EPI) (TR = 800 ms, TE = 37 ms, 2.0‐mm isotropic voxels, FoV = 208 mm, 72 sagittal slices, flip angle = 52°, bandwidth = 2290 Hz/Px, echo spacing = 0.58 ms, 495 volumes per session). Resting‐state fMRI were obtained with fast gradient‐echo EPI (TR = 3000 ms, TE = 30 ms, 3.0‐mm isotropic voxels, FoV = 216 mm, 47 sagittal slices, flip angle = 85°, bandwidth = 2240 Hz/Px, echo spacing = 0.51 ms, 100 volumes per session). All participants received extensive training on the tasks prior to scanning to ensure understanding and accurate task performance. Participants demonstrated correct performance outside of the scanner and after MRI positioning.

For task‐based scans, participants repeated the motor tasks interleaved with rest blocks in the MRI scanner (block duration = 12 seconds). Participants performed self‐paced left and right index finger tapping in alternating blocks for the hand task. For the vocal task, participants repeated the phonation of /i:/ (“ee”) during the task block. Each task scan session lasted 396 seconds. Participants completed one hand task session (6.6 minutes) and two vocal task sessions (13.2 minutes) to ensure a robust laryngeal activation pattern.^42^ For resting‐state scans, participants were instructed to remain still with eyes closed, staying awake and relaxed without thinking of anything specific. Each resting‐state scan lasted 300 seconds, and each participant completed four resting‐state runs (20 minutes total).

### MRI Processing

Task‐based and resting‐state fMRI data were preprocessed through an identical pipeline, previously described^43^, ^44^ and implemented using the personalized Brain Functional Sectors Cloud version 1.0.7 (Neural Galaxy Inc., Beijing, China). Each fMRI run was preprocessed separately to account for potentially run‐level signal differences. Preprocessing included discarding the first four volumes of each session for T1 stabilization, slice timing correction via SPM12 (Wellcome Center for Human Neuroimaging, London, UK), motion correction via FSL (FMRIB, Oxford, UK), normalization of global mean signal intensity, bandpass filtering (0.01–0.08 Hz), and regression of head motion and average signals from the whole brain, ventricles, and white matter. Individual cortical surfaces were registered to the *fsaverage6* template using FreeSurfer version 6.0 (MGH, Boston, MA, USA). Individual subcortical and cerebellar volumes were registered to a template (MNI152NLin6Asym) with a 2‐mm isotropic voxel size. A 6‐mm full‐width‐half‐maximum Gaussian smoothing kernel was then applied to both surface and volumetric data to improve the signal‐to‐noise ratio. The residual blood‐oxygen‐level‐dependent (BOLD) signals were used in the following analyses using custom MATLAB scripts (The MathWorks Inc., Natick, MA, USA).

### ROI Definition

#### Surface Task‐Based ROI

**FIG. 2:**
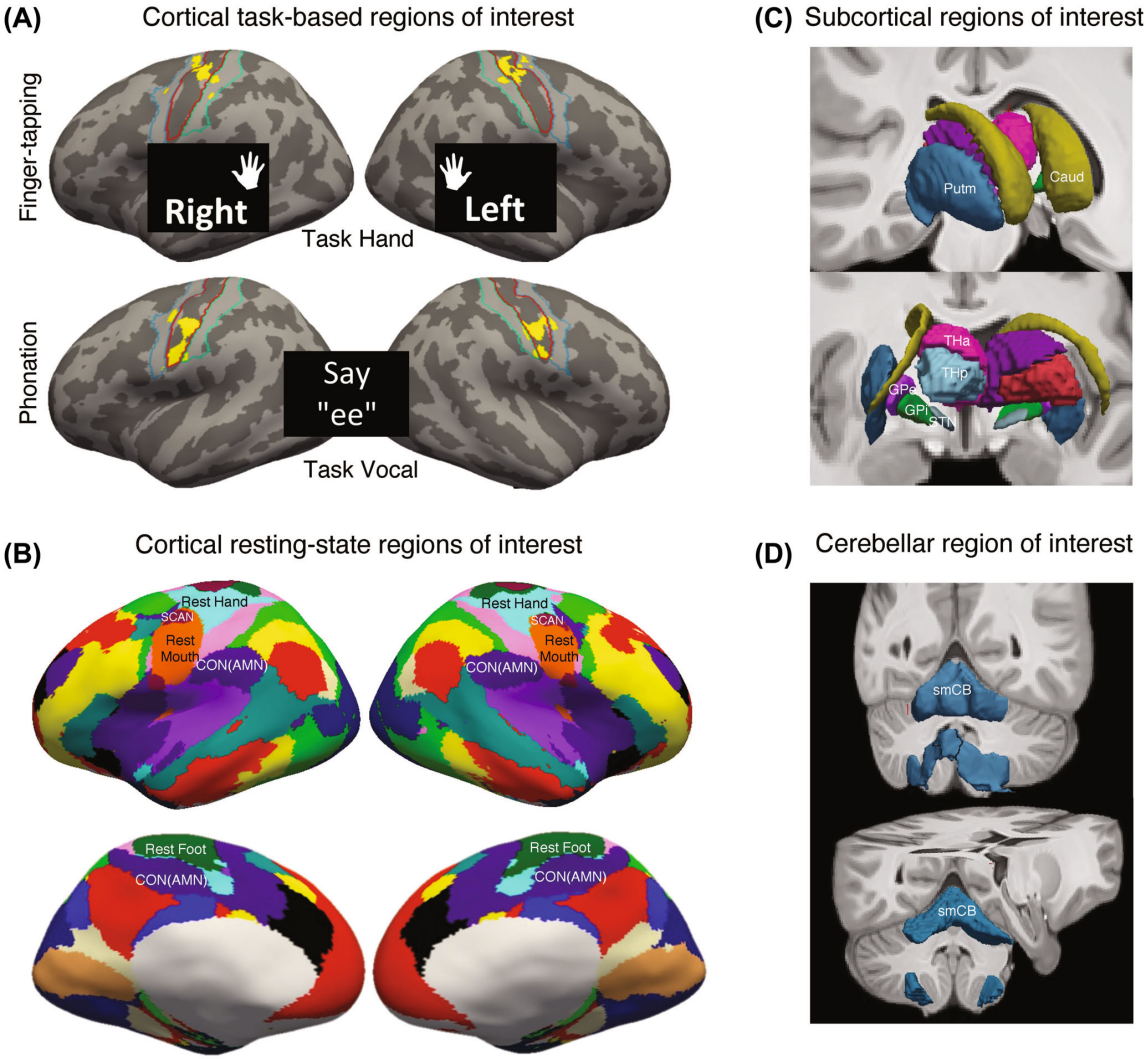
Surface and volumetric regions of interest (ROI). The affected effector regions (hand for focal hand dystonia [FHD], mouth/larynx for laryngeal dystonia [LD]) were defined from task and resting‐state scans for comparison. Group atlases were used for subcortical and cerebellar volumetric ROIs. (**A**) Task hand and task vocal ROIs are the peak activation regions (yellow) from finger‐tapping and phonation tasks, respectively, in an individualized manner (see Figs. S1 and S2). Task hand ROI on each hemisphere was separately generated from contralateral index finger‐tapping and combined, whereas bilateral task vocal ROI was from phonating/i:/. An a priori mask with pre‐ and postcentral gyrus (blue and tortoise outline) and central sulcus (red outline) was used to limit the task‐based effector ROI to the primary sensorimotor cortex (Destrieux et al., 2010). (**B**) The resting‐state parcellation algorithm divided the cortex into 18 nonoverlapping functional networks per hemisphere, which included rest hand, rest mouth, SCAN, CON(AMN), and (rest) foot (Gordon et al., 2017, 2023). This iterative parcellation algorithm adjusted individual network boundaries based on *k*‐means clustering (Fig. S3). All surface ROIs were on the *fsaverage6* surface. (**C**) Subcortical volumetric ROIs were obtained from atlases (Pauli et al., 2018, Tian et al., 2020) in MNI152 space and resampled to an isotropic voxel size of 2 mm. (**D**) Sensorimotor part of the cerebellum was obtained (Buckner et al., 2011) in MNI152 space and resampled to an isotropic voxel size of 2 mm. AMN, action‐mode network; Caud, caudate; CON, cingulo‐opercular network; GPe, globus pallidus externus; GPi, globus pallidus internus; Putm, putamen; SCAN, somato‐cognitive action network; smCB, sensorimotor cerebellum; STN, subthalamic nucleus; THa, anterior thalamus; THp, posterior thalamus. [Color figure can be viewed at wileyonlinelibrary.com]

**FIG. 3:**
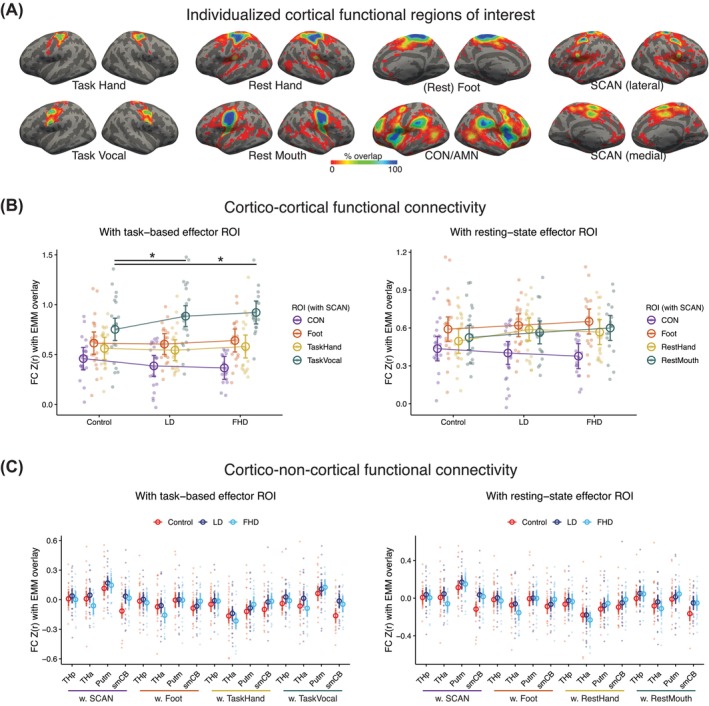
Individualized cortical regions of interest (ROIs) and linear model results. (**A**) Individualized cortical ROIs are shown on the *fsaverage6* surface, with the color scale indicating percentage overlap for all subjects in our study after quality control (N = 57 for task hand, N = 56 for task vocal, N = 61 for resting‐state parcellation). Foot ROI was included as an active control region. (**B**) Cortical functional connectivity (FC) changes involving SCAN were tested with both task‐derived and resting‐state ROIs. With task‐based effector ROI (left), SCAN demonstrated a significant interaction effect for both laryngeal dystonia (LD) and focal hand dystonia (FHD) groups compared to control (*P* = 0.048, *P* = 0.017, respectively). This interaction was driven by SCAN's higher connectivity with task vocal ROI and lower connectivity with CON (ie, AMN) in focal dystonia groups. No significant dystonia‐dependent effects were found with resting‐state effector ROIs (right). (**C**) We tested if cortical effector regions and/or SCAN showed FC changes with closely synapsed basal ganglia and cerebellar circuitry. Task‐based and resting‐state cortical ROIs were included in separate linear models. We did not find significant cortico‐subcortical or cortical‐cerebellar connections related to LD or FHD. For all linear model results, raw FC values after *r*‐to‐*z* transform were plotted with an overlay of estimated marginal mean (EMM), which accounts for covariates (age, sex, symptom duration, and mean relative motion during resting‐state scan). All error bars indicate 95% confidence interval of the EMM. AMN, action‐mode network; Caud, caudate; CON: cingulo‐opercular network; GPe, globus pallidus externus; GPi, globus pallidus internus; Putm, putamen; SCAN: somato‐cognitive action network; smCB, sensorimotor cerebellum; STN, subthalamic nucleus; THa, anterior thalamus; THp, posterior thalamus.

The motor task activation areas were used to determine the task‐based ROIs (hand and vocal) on an individual level. For each run, task‐related regressors (ie, left or right finger‐tapping vs. rest, or phonation vs. rest), which were convolved with a canonical hemodynamic response function, were added in the general linear model (GLM) of the subject‐level task data with six parameters of motion regressor. Vertex‐wise beta values associated with the motor tasks (averaged between the two vocal task runs) were then *z* score transformed. Task‐based ROIs were defined as the top 10% of *z* scores in the primary sensorimotor region (precentral and postcentral gyri and central sulcus in Destrieux atlas^45^) or *z* score >2.33 (one‐tailed vertex‐wise *P*‐value < 0.01, uncorrected), whichever was greater. The percentage threshold was determined to ensure that vocal ROI matched the established ventral–dorsal laryngeal pattern^1^, ^46^ and also applied to hand ROI for signal comparability (Figs. S1 and S2). For hand task, separate left and right finger‐tapping regressors were used to define the hand ROI on the contralateral hemisphere. For the vocal task, a single phonation regressor determined vocal ROI bilaterally (Fig. 2A). The resulting task‐based ROIs were quality controlled based on (1) task scan mean relative motion <0.4 mm (identical to the motion threshold of resting‐state scan) and (2) arbitrarily >20 vertices surviving the *z* score thresholding in each hemisphere to ensure sufficient data for BOLD signal averaging. For subjects' task data that had scan error (incomplete scan file, corrupt task trigger timing) or failed quality control above (hand task: 4 scan error +2 below‐threshold ROI; vocal task: 3 scan error +1 excessive motion +3 below‐threshold ROI) (Figs. S1 and S2), hand and mouth ROIs from the individualized resting‐state parcellation were used instead to maintain power, given the general spatial and functional similarity between task‐based and resting‐state effector regions (Fig. 3A; Figs. S6 and S7).

#### Surface Resting‐State ROI

The residual BOLD signals after preprocessing were concatenated across four runs to generate individualized resting‐state parcellations using the same pipeline previously described.^44^ An 18‐network group atlas was constructed by combining SCAN^1^ with a 17‐network parcellation that distinguished hand, mouth, and foot regions^47^ (Fig. 2B). This group atlas was used to initialize an iterative *k*‐means clustering algorithm that adjusted the network boundaries based on individual resting‐state data to minimize the heterogeneity within each functional network. The SCAN region was identified with an interdigitated topography along the primary motor cortex as expected. Effector regions (rest hand, rest mouth, and foot) and CON, also known as the action‐mode network (AMN),^48^ were also obtained (Fig. S3).

#### Volumetric ROI

Subcortical gray matter and cerebellum ROIs (Fig. 2C,D) were generated based on previous atlases. Basal ganglia ROIs included caudate, putamen, globus pallidus externus (GPe), globus pallidus internus (GPi), and subthalamic nucleus (STN).^49^ The thalamus was divided into anterior and posterior segments.^50^ Sensorimotor region of the cerebellum was defined using a seven‐network atlas.^31^ All volumetric atlases were conformed to the aforementioned MNI152‐2‐mm space.

### Functional Connectivity Definition

Given the strong functional coupling of bilateral homologs in resting‐state scans, we combined all ROIs across both hemispheres. Resting‐state functional connectivity (FC) was computed as the zero‐lag Pearson's correlation in the average BOLD time series between ROIs. A Fisher's *r*‐to‐*z* transform was then performed for later statistical testing.

### Statistical Analysis

We performed three separate linear models with the following formula to investigate the interactions between dystonia group and FC between ROI pairs, accounting for covariates (ie, age, sex, symptom duration, and mean relative motion during resting‐state scan), given the difference between groups (Table 1), which may be influenced by the different incidence rates between sexes for LD and FHD.^51^, ^52^

FC~group×ROIpair+age+sex+symptom duration+mean relative motion

Linear model 1 tests if SCAN demonstrates dystonia‐specific changes with functionally related cortical networks (effector regions and CON/AMN) (Fig. 1B). Linear model 2 tests if primary sensorimotor networks (effector regions and SCAN) show dystonia‐specific changes with closely synapsed subcortical and cerebellar structures (thalamus, putamen, and sensorimotor cerebellum).^1^, ^34^, ^53^, ^54^, ^55^ For linear model 1 and 2, cortical effector ROIs defined with task versus resting‐state data were included in separate models for comparison. Linear model 3 was performed to explore if any functional connection within the subcortex and cerebellum revealed dystonia‐dependent changes. Based on overlapping patterns in the results, we conducted exploratory *post‐hoc* analyses that combined LD and FHD participants into a single focal dystonia group. No multiple comparison correction was performed on the linear model estimates. Visual inspection of residuals and Breusch‐Pagan tests were performed for each linear model to confirm homoscedasticity. All statistical analyses were performed in R (R Foundation for Statistical Computing, Vienna, Austria) in RStudio (Posit Software, Boston, MA, USA).

## Results

### LD and FHD Share Abnormal Cortical Connectivity Involving SCAN

In the assessment of cortical connectivity differences, LD and FHD demonstrated altered SCAN connectivity (*P* = 0.048, *P* = 0.017, respectively) compared to controls. Specifically, the effect was driven by SCAN's higher connectivity with task vocal and simultaneously lower connectivity with CON. In the same model with task‐based ROI, the connectivity between CON and SCAN was also significantly lower than those of task vocal‐SCAN and foot‐SCAN across all groups (*P* < 0.001, *P* = 0.038, respectively) (Fig. 3B left, *F*_(15,228)_ = 7.69, *P* < 0.001, *R* ^2^_adj_ = 0.29, *P*_Breusch‐Pagan_ = 0.083) (Table S1), suggesting that SCAN may be more closely linked to effector regions than CON on average, despite individual differences reported (Fig. S2 in^1^). In the resting‐state effector ROI analysis (Fig. 3B right, *F*_(15,228)_ = 2.83, *P* < 0.001, *R* ^2^_adj_ = 0.10, *P*_Breusch‐Pagan_ = 0.51), no dystonia‐dependent effects were found (Table S2).

### No LD‐ or FHD‐Specific Changes Were Observed in Subcortical or Cerebellar Pathways

In the subcortical and cerebellar analysis, there were no significant dystonia‐related differences compared to control (Fig. 3C, Fig. S4). The model details were as follows: model 2 with task‐based ROI (Fig. 3C left) *F*_(51,924)_ = 4.38, *P* < 0.001, *R* ^2^_adj_ = 0.15, *P*_Breusch‐Pagan_ = 0.098 (Table S3); model 2 with resting‐state ROI (Fig. 3C right) *F*_(51,924)_ = 4.21, *P* < 0.001, *R* ^2^_adj_ = 0.14, *P*_Breusch‐Pagan_ = 0.37 (Table S4); model 3 within noncortical ROI (Fig. S4) *F*_(30,518)_ = 47.38, *P* < 0.001, *R* ^2^_adj_ = 0.72, *P*_Breusch‐Pagan_ = 0.065 (Table S5).

### There Is Shared Asynchronization Between SCAN and Sensorimotor Cerebellum in Focal Dystonia

**FIG. 4:**
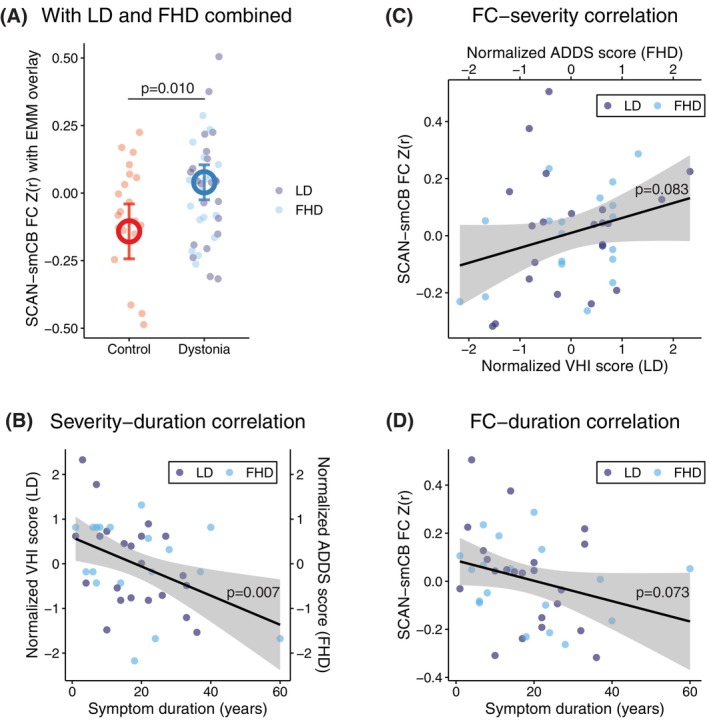
Exploratory investigation of the functional connectivity between SCAN and sensorimotor cerebellum in focal dystonia. (**A**) The dystonia group combining laryngeal dystonia (LD) and focal hand dystonia (FHD) showed significantly different FC (*P* = 0.010) compared to controls. Specifically, there was a weak negative FC between SCAN and sensorimotor cerebellum in control subjects, which diminished to around zero in the combined dystonia group. This may suggest an asynchronization of the cortico‐cerebellar sensorimotor connection with a loss of tonic inhibition at rest in focal dystonia. (**B**) Dystonia severity was negatively correlated with symptom duration (*P* = 0.007). Severity measures of dystonia (VHI and ADDS) were normalized (mean‐centered and divided by the standard deviation) to allow being pooled for correlation tests. Higher normalized severity measure indicates greater symptoms. (**C**) There was a trend toward positive correlation of SCAN‐cerebellum FC against severity (*P* = 0.083) and (**D**) a trend toward negative correlation against symptom duration (*P* = 0.073). ADDS, Arm Dystonia Disability Scale; EMM, estimated marginal mean; FC, functional connectivity; SCAN, somato‐cognitive action network; smCB, sensorimotor cerebellum; VHI, Voice Handicap Index. [Color figure can be viewed at wileyonlinelibrary.com]

Given the similarity in FC distribution between LD and FHD (Fig. 3C) and the marginal interaction terms involving SCAN and sensorimotor cerebellum (*P* = 0.088 for FHD, *P* = 0.11 for LD, Table S3), we combined both subtypes for increased statistical power and to explore potentially shared functional changes in focal dystonia.^30^, ^56^ A post‐hoc linear model was performed with the following formula (Fig. 4A, *F*_(5,55)_ = 2.82, *P* = 0.0244, *R* ^2^_adj_ = 0.13, *P*_Breusch‐Pagan_ = 0.75) (Table S6).

$${\mathrm{FC}}_{\text{SCAN}-\text{smCB}}\sim {\text{group}}_{\text{combined}}+\mathrm{age}+\mathrm{sex}+\text{symptom duration}+\text{mean relative motion}$$

The combined dystonia group showed significantly different connectivity between SCAN and sensorimotor cerebellum compared to controls (*P* = 0.010). To further understand if this SCAN‐cerebellum FC was linked to dystonia pathophysiology, we tested its correlation with symptom duration and severity. Severity measures of dystonia (VHI and ADDS) were normalized (mean‐centered and divided by the standard deviation) to allow for correlation test pooling. Higher normalized severity measures indicated greater symptoms. Dystonia severity was negatively correlated with symptom duration (*P* = 0.007, Fig. 4B). There was a weak trend toward positive correlation of SCAN‐cerebellum FC against severity (Fig. 4C, *P* = 0.083) and towards negative correlation against symptom duration (Fig. 4D, *P* = 0.073).

## Discussion

For the first time, our study systematically tested whether SCAN versus dystonic effector regions demonstrate abnormal functional connectivity in two types of focal dystonia. Our results suggest that (1) SCAN is involved in focal dystonia pathology regardless of the affected body part (hand or larynx), and (2) dystonia‐related SCAN changes include higher connectivity with task‐based effector regions with concomitantly lower connectivity with CON and asynchronization with the sensorimotor cerebellum at rest. This is consistent with our hypothesis 1 (SCAN is associated with focal dystonia pathology) but contrary to our hypothesis 2 (SCAN is more relevant in the axial LD rather than the appendicular FHD). There is partial support for hypothesis 3 (focal dystonia dysfunction can be localized in sensorimotor control pathways in a dystonic body part–specific manner), given the group difference involving SCAN and sensorimotor cerebellum, but the functional changes were not specific to the dystonic body part.

### Individualized Cortical ROIs

Defining individual brain networks beyond group atlases has been challenging in focal dystonia, as prior research identified spatial changes in dystonic effector networks,^14^, ^35^, ^36^, ^38^ perhaps due to sensorimotor reorganization.^57^, ^58^ These individual variations in network boundaries may particularly impact the accuracy of more fine‐grained networks such as SCAN. We overcame these challenges by adjusting cortical ROI boundaries based on individual functional data.^44^ The limited group‐level overlap (Fig. 3A) reinforces the value of our approach.

Additionally, the dystonia‐related changes detected with task‐based, but not resting‐state, effector ROIs (Fig. 3B left vs. right) may suggest that the individual variability in motor tasks is not fully captured in resting‐state parcellation. If so, future dystonia research may need to move beyond resting‐state data alone. However, rather than a fundamental difference between task versus resting‐state approaches, the difference in ROI results might instead reflect the greater specificity of a laryngeal network elicited via phonation, as opposed to a more general mouth region seeded in our resting‐state atlas (Fig. 2B). It remains unknown if initializing the resting‐state parcellation with a laryngeal network within the mouth region may yield results comparable to the phonation task.

### Cortical Functional Changes

One crucial unresolved question in focal dystonia is whether the pathophysiology differs depending on the body part affected.^59^, ^60^ Few empirical studies have included focal dystonia affecting different effectors, and the results have been mixed. For example, some studies showed convergence in left insula abnormality in both LD and FHD but pointed to different regions depending on whether functional or structural data were used.^27^, ^30^, ^61^ With resting‐state fMRI, studies found widespread changes across the brain or localized changes at the right inferior parietal lobule.^26^, ^29^ Our novel approach probing the basal ganglia‐thalamo‐cortical and cerebello‐thalamo‐cortical pathways in human focal dystonia may complement graph theory and ROI‐based methods^24^, ^25^, ^57^, ^62^ to bridge with the extensive circuit‐level findings from animal literature.^53^, ^63^

The cortico‐cortical abnormality involving the mouth/larynx region (higher SCAN‐task vocal connectivity with lower SCAN‐CON connectivity compared to controls) shared in LD and FHD (Fig. 3B) may suggest a dystonia‐related predilection rather than a symptom‐causing biomarker, as FHD subjects did not experience laryngeal symptoms. Clinically, it is known that focal dystonia affecting one body part may spread to other isolated body parts,^60^ pointing to common central pathology beyond the involved muscles. Given the role of SCAN in mediating goal‐directed cognition (CON)^48^ and fine motor control (eg, effector‐specific task vocal),^1^ the shifting SCAN connectivity may explain the greater difficulty executing a motor plan in a top‐down manner in focal dystonia.

Identifying altered SCAN‐CON function in focal dystonia may also shed light on past research. The role of SCAN in mediating high‐level action intention from CON may be particularly relevant for task specificity in focal dystonia. Both LD and FHD are task‐specific: dystonic symptoms only manifest during specific, highly practiced tasks (eg, speaking, writing, playing the hand drum) but not during other movements (eg, singing, buttoning shirts). The decreased SCAN‐CON connectivity may signify less‐effective relay of intention. Perhaps supporting this idea, FHD patients were found to have decreased structural connectivity in likely key nodes of CON (bilateral insula and right paracentral lobule); however, the SCAN‐CON relevance had not yet been determined.^30^

Additionally, isolating SCAN as a distinct network within the primary motor cortex facilitates a more straightforward interpretation of pathophysiology. For example, our group previously identified an activated cluster in the superior motor cortex that correlated with a cortical inhibition measure in LD but not healthy controls.^42^ A mechanistic interpretation was difficult at the time because the cluster was outside the laryngeal motor cortex in what appeared to be the trunk motor region. A similar cluster in the superior motor cortex has also been reported in LD during syllable and whimper production.^35^ It is compelling that both studies may have observed the engagement of SCAN in regulating dystonia physiology.

A potential confound to our cortico‐cortical interaction effect (Fig. 3B left) is that task vocal ROI may overlap with SCAN to different extents in dystonia and control subjects, thus inflating the functional connectivity between task vocal and SCAN in a dystonia‐dependent manner. This is unlikely to fully account for the interaction effect, however, because the overlap was low (group mean overlap <15% of the individual SCAN area), and no significant group difference was found (Fig. S5, also see peak location difference in Supplementary Material). Additionally, the positive findings were reinforced given that the FC changes were not observed in our nonaffected control ROI (foot).

Several reasons may explain why we did not find similar dystonia‐related changes in the hand region. First, unlike laryngeal control, which is bilateral through the corticobulbar tract, hand control is heavily lateralized via the corticospinal tract, and its abnormal activity may therefore be difficult to detect after averaging over bilateral ROIs. Second, our phonation task may be more symptom‐inducing than finger‐tapping, leading to more accurate estimation of the affected functional network in LD rather than FHD. Third, the task quality difference may be compounded by greater heterogeneity in FHD phenotypes, as our subjects had different hand (left, right, bilateral) and finger/wrist involvement.

### Cerebellar Functional Changes

Finding key pathological functional alterations can update our disease categorization^7^ and inform treatment approaches that allow for reconfiguration of the pathological brain networks.^64^, ^65^, ^66^, ^67^, ^68^, ^69^ The SCAN‐cerebellum connection in our exploratory model combining LD and FHD groups may be such a candidate (Fig. 4A). Crucially, there was a weak negative functional connectivity between SCAN and sensorimotor cerebellum in healthy controls, which diminished to around zero (indicating asynchronization) in the combined dystonia group based on the estimated marginal mean.

This finding reinforces and extends seed‐based resting‐state changes, previously reported in cervical dystonia and blepharospasm. With a seed at the output nucleus of the cerebellum (dentate), a “loss of anticorrelation” was coarsely observed in the primary sensorimotor cortex.^25^ Our results not only validate this functional asynchrony in focal dystonia affecting other body parts (larynx and hand) but also isolate the unique role of SCAN within the primary sensorimotor cortex. Nevertheless, further work is needed to determine if non–task‐specific focal dystonia also demonstrates similar cortical interactions involving CON, which is closely related to movement intention.^48^

To investigate whether this SCAN‐cerebellum connectivity may be relevant for pathophysiology in our cohort, we reasoned that the extent of the FC deviation away from the normal (weak negative FC) should positively correlate with symptom severity. This was weakly supported by the correlation result (Fig. 4C). Alternatively, symptom duration was negatively correlated with disease severity (Fig. 4B), possibly suggesting that the brain may have undergone neuroplastic changes that ameliorated the pathology over time. This amelioration of dystonia symptoms was marginally associated with lowered SCAN‐cerebellum FC (ie, a return to weak negative connectivity similar to controls) (Fig. 4D). It is, therefore, plausible that SCAN‐cerebellum asynchronization reflects pathological rather than compensatory changes across dystonic body parts or task specificity. This is corroborated by lesion network mapping that linked SCAN and likely sensorimotor cerebellum to dystonic symptoms.^19^

The weak negative connectivity between the cerebellum and SCAN may reflect a form of healthy tonic inhibition at rest, which was disrupted with focal dystonia onset. This loss of inhibition in cerebello‐thalamo‐cortical pathway was consistent with prior neurophysiological findings mainly in FHD (cf.^70^)^71^, ^72^, ^73^, ^74^ and could be a result of the decreased white matter integrity in dentate‐rubro‐thalamic tract, which was found in cervical dystonia.^75^ However, the correlational nature of functional connectivity makes it challenging to infer the sensorimotor cerebellum's relative afferent versus efferent contribution to the asynchronization. Interestingly, a recent preclinical study found that this cerebello‐thalamo‐cortical pathway was responsible for learned, context‐dependent movement initiation, which may further link to task specificity.^76^ Our findings may support novel treatment targets, such as the cerebellum, in the context of emergent clinical trials.^77^, ^78^, ^79^

It remains to be answered why, if SCAN‐cerebellum asynchronization is pathological across dystonic body parts, DBS‐based and lesion‐based network mapping inconsistently identify SCAN and cerebellum with varying CON involvement.^19^, ^20^ One possibility is that SCAN disruption causes dystonia across body parts, but CON targeting improves axial dystonia more. If the decreasing SCAN‐CON connectivity we observed is predispositional, DBS may alleviate dystonic symptoms by upregulating CON in a compensatory manner. The absence of SCAN in DBS network mapping^20^ does not preclude potential modulatory effects on SCAN, because seeding in a normative functional connectome may only have enough signal strength to detect the immediate downstream effect.

## Limitations

There are limitations in the current study. Our limited sample size without power analysis may have led to reduced statistical power that failed to detect functional changes, for example, in basal ganglia pathways. The self‐reported disease severity measures had poor sensitivity, and we did not obtain dystonia‐related genetic information, which could introduce heterogeneity.^5^ Given our relatively small sample size, we did not differentiate between different phenotypes within LD (adductor vs. abductor) and FHD (handedness, finger/wrist involvement). In combining bilateral ROIs, we omitted laterality, which may be relevant even for LD.^80^ Our results on SCAN‐cerebellum connectivity across LD and FHD remain correlational and need further testing. Finally, our volumetric ROIs may be too coarse to detect localized changes, for example, in the centromedian nucleus of the thalamus.^1^

Focal dystonia is a debilitating disease with unclear pathophysiology and limited treatment. The role of SCAN in coordinating among cognitive intention, physiologic control, and motor execution may be particularly relevant for task‐specific focal dystonia, though the SCAN‐cerebellum asynchronization appears shared regardless of task‐specificity or the dystonic body part. Our results show that SCAN is uniquely associated with dystonia dysfunction beyond the dystonic effector regions, potentially updating our understanding of pathophysiology and inspiring treatment targets.

## Author Roles

(1) Research Project: A. Conception, B. Organization, C. Execution; (2) Statistical Analysis: A. Design, B. Execution, C. Review and Critique; (3) Manuscript Preparation: A. Writing of the First Draft, B. Review and Critique.

Y.W.: 1A, 1C, 2A, 2B, 3A.

B.H.: 1B, 1C, 3B.

J.R.: 2C, 3B.

M.C.: 1A, 1B, 1C, 3B.

W.Z.: 2B, 3B.

D.H.: 2B, 3B.

S.L.: 2C, 3B.

H.L.: 2C, 3B.

T.J.K.: 1A, 1B, 1C, 2C, 3B.

## Supporting information


**Data S1.** Supplementary material.

**Data S2.** STROBE reporting checklist.

## Data Availability

Data and code used for statistical analysis and visualization are available at https://osf.io/nmdka/.
