# Supplementary material for: Somato‐Cognitive Action Network in Focal Dystonia
Source: Mov Disord. 2025 Aug 28;40(11):2331–43. doi: 10.1002/mds.70021 (PMC12661636; doi:10.1002/mds.70021)
Supplement: Supplementary file 1 — Data S1. Supplementary material. [file MDS-40-2331-s002.docx]

**Supplementary material: Somato-cognitive action network in focal dystonia**

Yuchao Wang, Baothy Huynh, Jianxun Ren, Mo Chen, Wei Zhang, Dan Hu, Shasha Li, Hesheng Liu, Teresa J. Kimberley

**Distinguishing Task Vocal vs. SCAN**

Notably, our phonation task generated a ventral and dorsal pattern grossly within the Rest Mouth network, consistent with the laryngeal motor network (cf. ^1^).^2–4^ Our Task Vocal ROI also consisted of an area in the superior sensorimotor cortex bilaterally (left hemisphere: 18% peak overlap at (-18.1, -24.9, 61.7) in RAS coordinates on the *fsaverage6* surface, same below, right hemisphere: 29% peak overlap at (19.2, -25.4, 57.5)). This region was previously thought to be the trunk or abdominal area,^3,4^ but overlapped with SCAN, which likely reflected the involvement of SCAN in controlling breathing for vocalizing.^2^ Despite the partial overlap, SCAN and Task Vocal ROI are distinct functional networks, with the Task Vocal (laryngeal) ROI being located more inferiorly and anteriorly to the SCAN in the ventral primary sensorimotor cortex (left SCAN: 98% peak overlap at (-35.0, -15.1, 42.0), right SCAN: 94% peak overlap at (36.7, -12.7, 40.3); left Task Vocal: 82% peak overlap at (-50.0, -9.5, 45.2), right Task Vocal: 73% peak overlap at (50.5,-5.7, 45.0)).

1. Simonyan, K., Ostuni, J., Ludlow, C. L. & Horwitz, B. Functional But Not Structural Networks of the Human Laryngeal Motor Cortex Show Left Hemispheric Lateralization during Syllable But Not Breathing Production. *J. Neurosci.* **29**, 14912–14923 (2009).

2. Gordon, E. M. *et al.* A somato-cognitive action network alternates with effector regions in motor cortex. *Nature* **617**, 351–359 (2023).

3. Chen, M. *et al.* Transcranial magnetic stimulation and functional magnet resonance imaging evaluation of adductor spasmodic dysphonia during phonation. *Brain Stimulat.* **13**, 908–915 (2020).

4. Correia, J. M., Caballero-Gaudes, C., Guediche, S. & Carreiras, M. Phonatory and articulatory representations of speech production in cortical and subcortical fMRI responses. *Sci. Rep.* **10**, 4529 (2020).


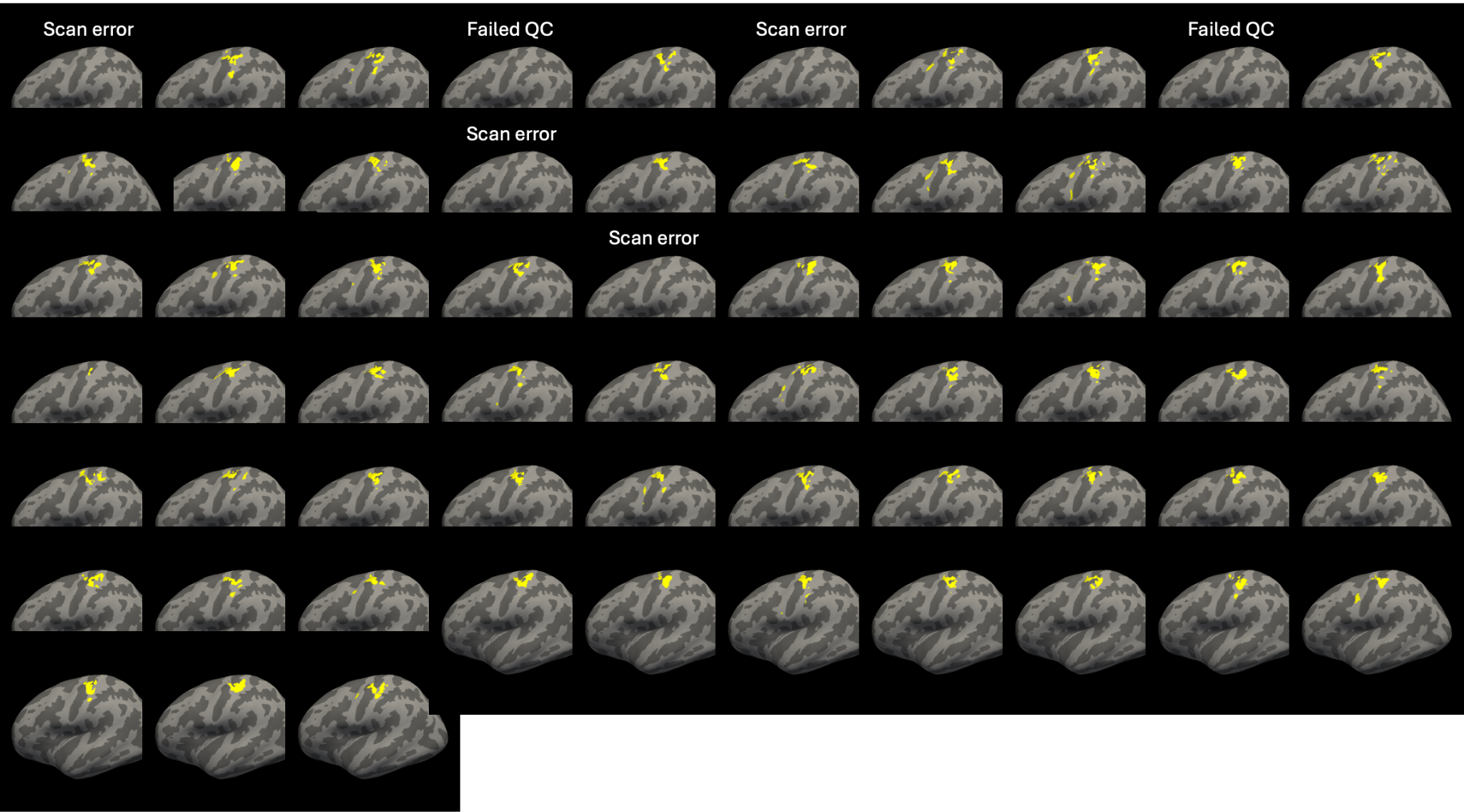

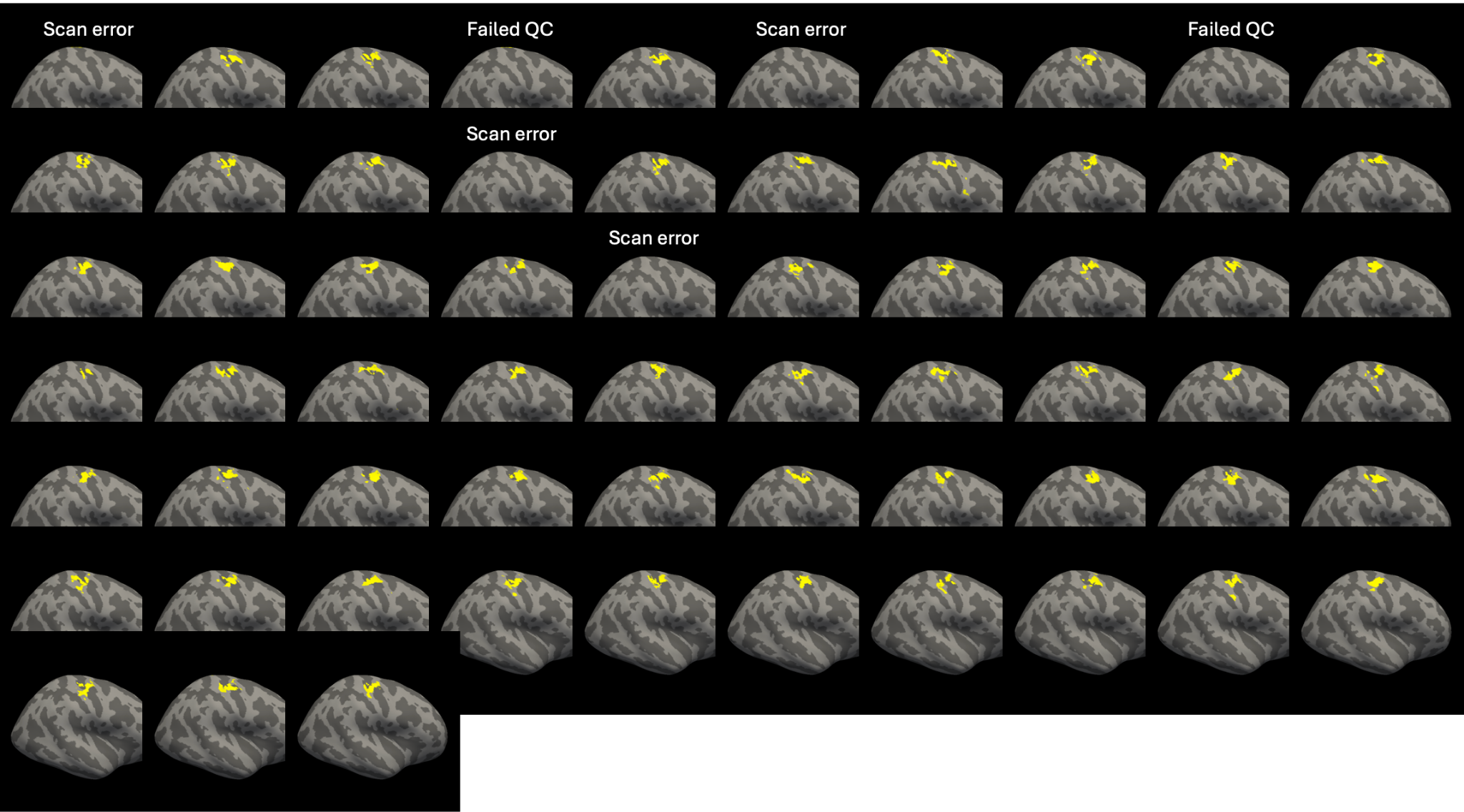


**Supplementary Figure 1: Individualized Task Hand ROI.**


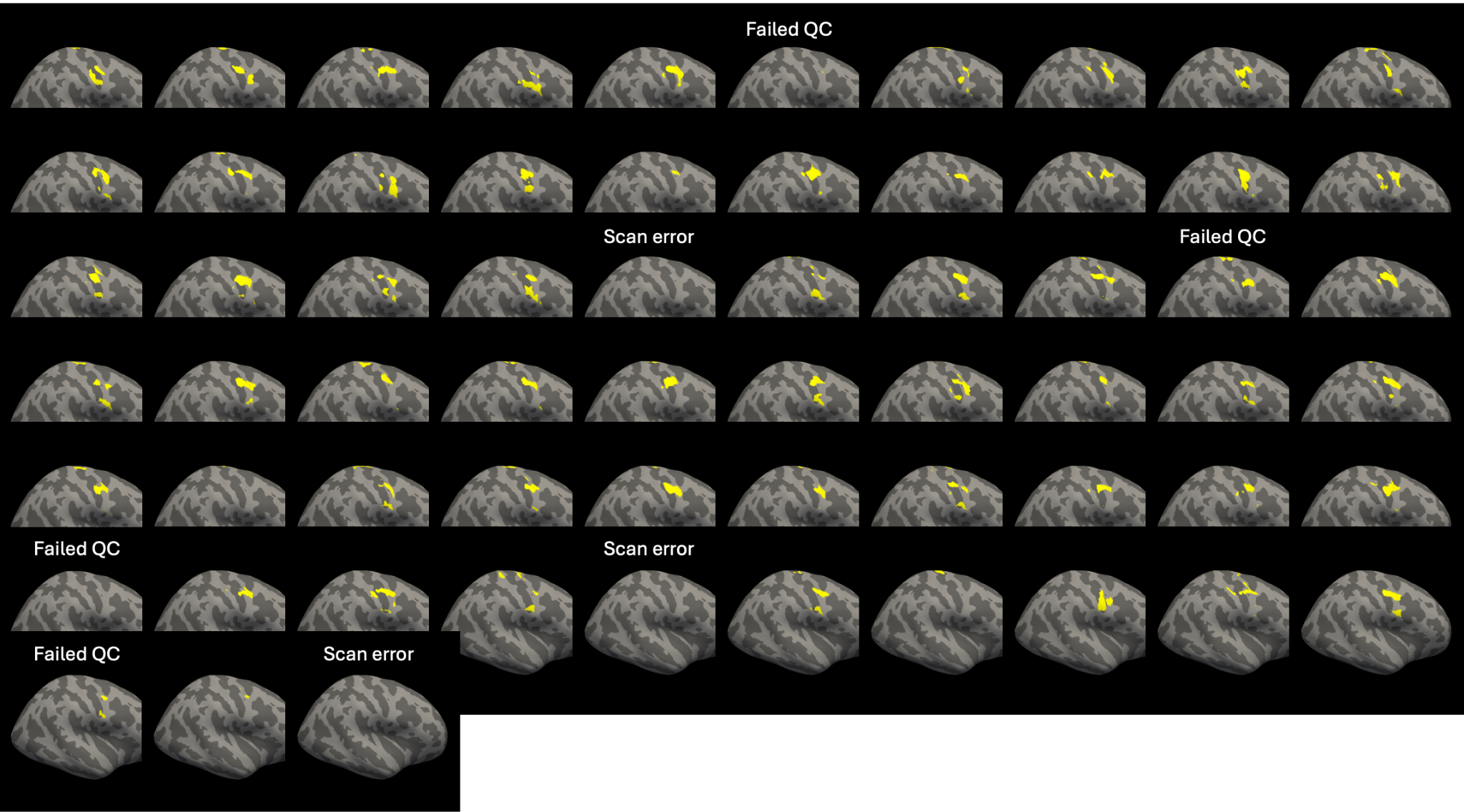

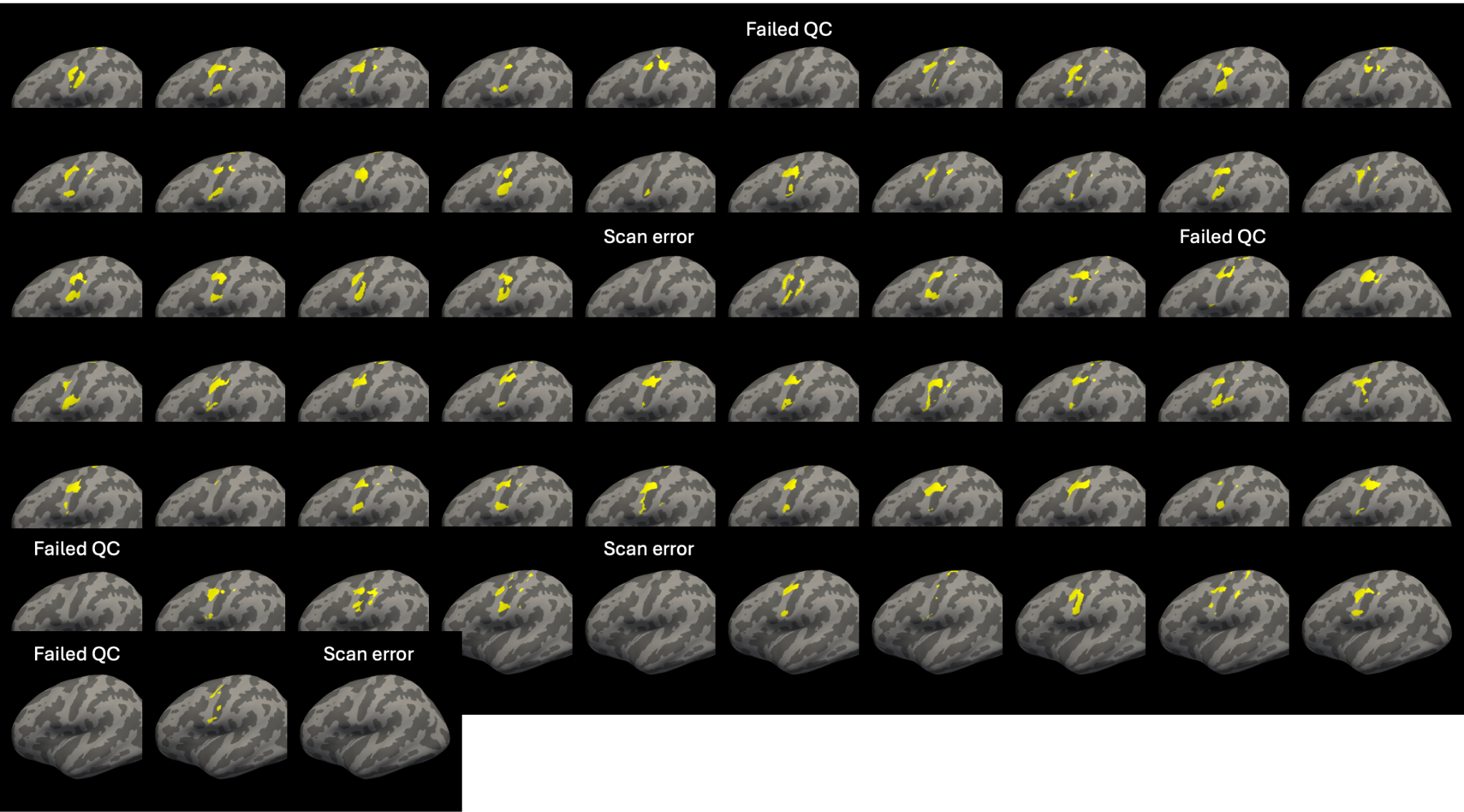
**Supplementary Figure 2: Individualized Task Vocal ROI.**


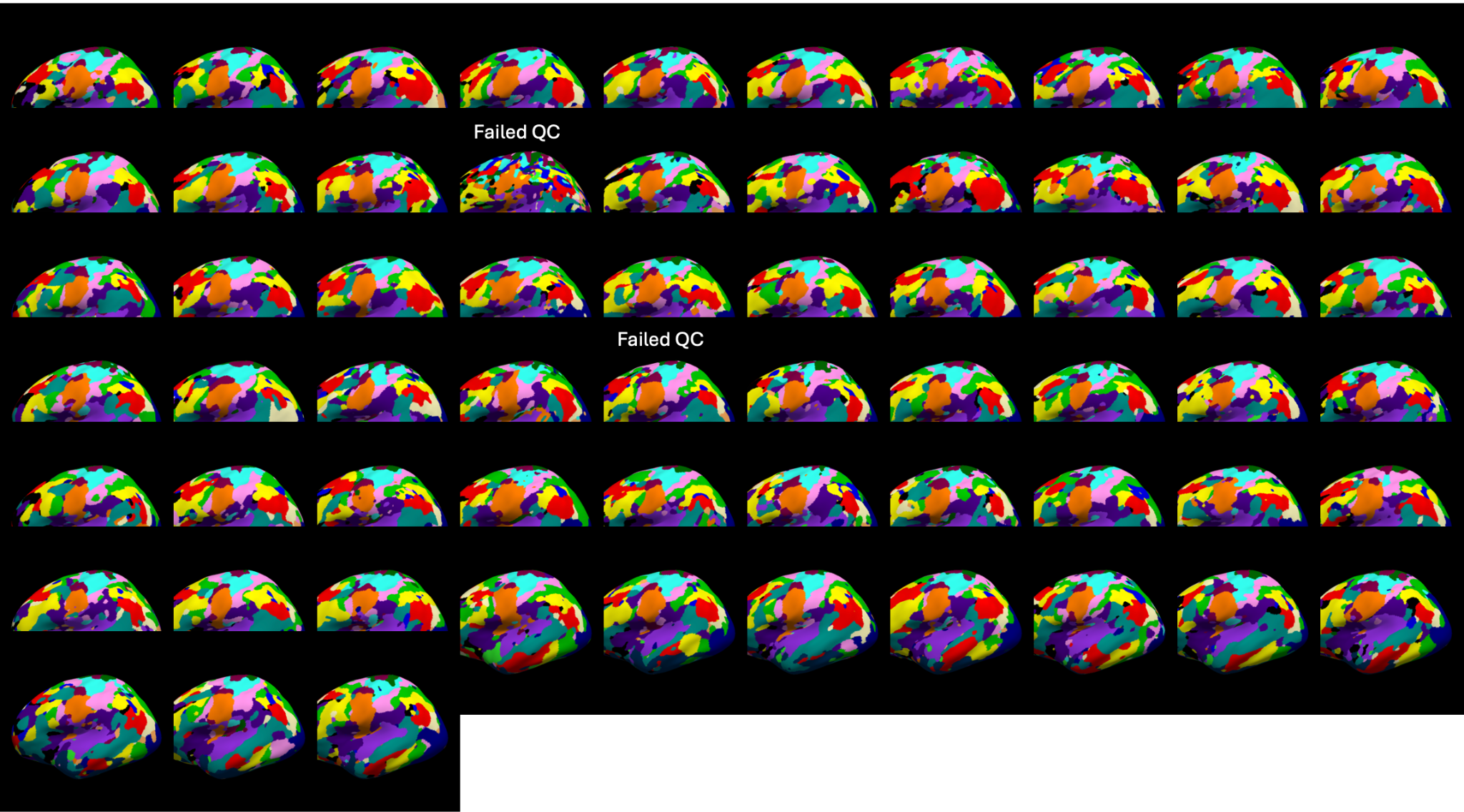


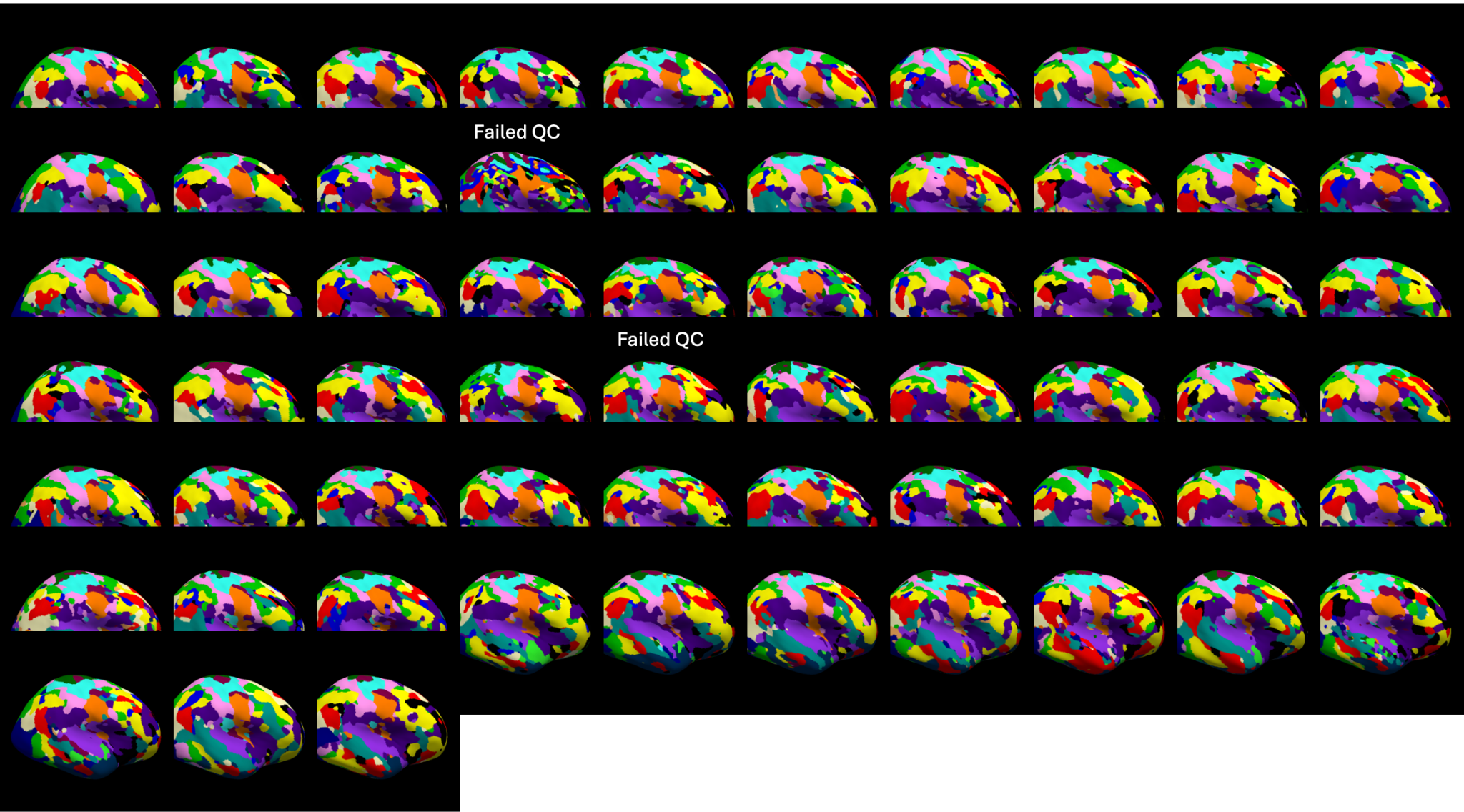


**Supplementary Figure 3: Individualized resting-state parcellation results.** Rest Hand is in cyan, Rest Mouth in orange, Foot in dark green, SCAN in marron, and CON(AMN) in dark purple. SCAN: somato-cognitive action network. CON: cingulo-opercular network. AMN: action-mode network.

**Supplementary Figure 4: No significant dystonia-dependent effects were found among subcortical and cerebellar ROIs (linear model 3).** Raw FC values after r-to-z transform were plotted with an overlay of estimated marginal mean (EMM), which accounts for covariates (age, sex, symptom duration, and mean relative motion during resting-state scan). All error bars indicate 95% confidence interval of the EMM. SCAN: somato-cognitive action network. CON: cingulo-opercular network. AMN: action-mode network. Caud: caudate. Putm: putamen. GPe: globus pallidus externus. GPi: globus pallidus internus. STN: subthalamic nucleus. THa: anterior thalamus. THp: posterior thalamus. smCB: sensorimotor cerebellum.


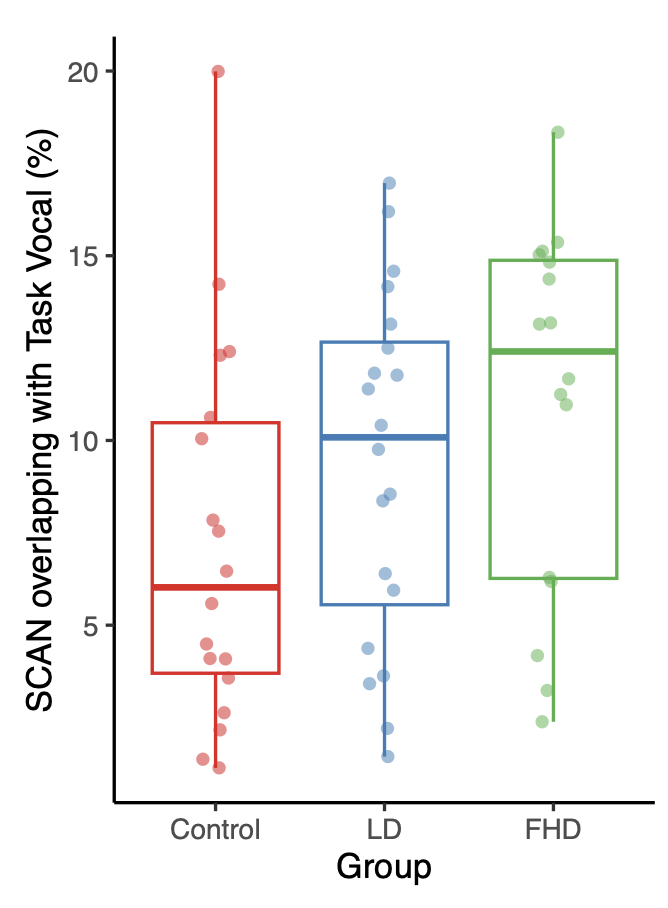


**Supplementary Figure 5: Overlap between SCAN and Task Vocal.** No significant group difference was found (one-way ANOVA, *F*(2,51)=2.44, *p*=0.098) though a trend was observed with greater overlap between SCAN and Task Vocal in focal dystonia groups.


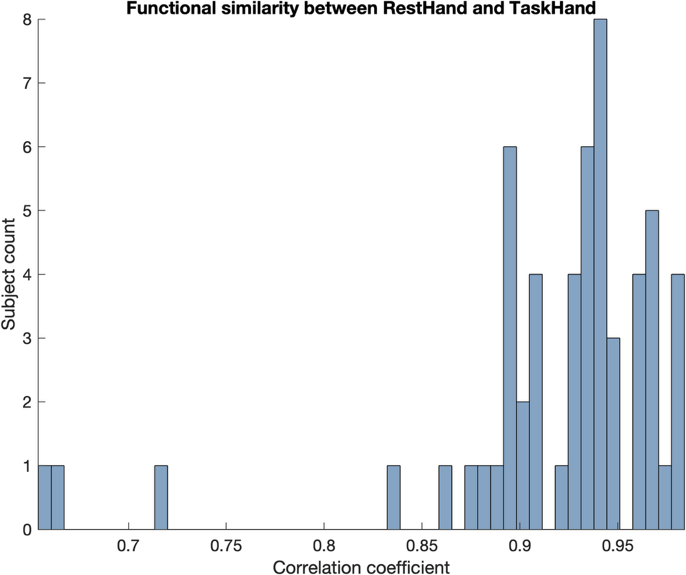

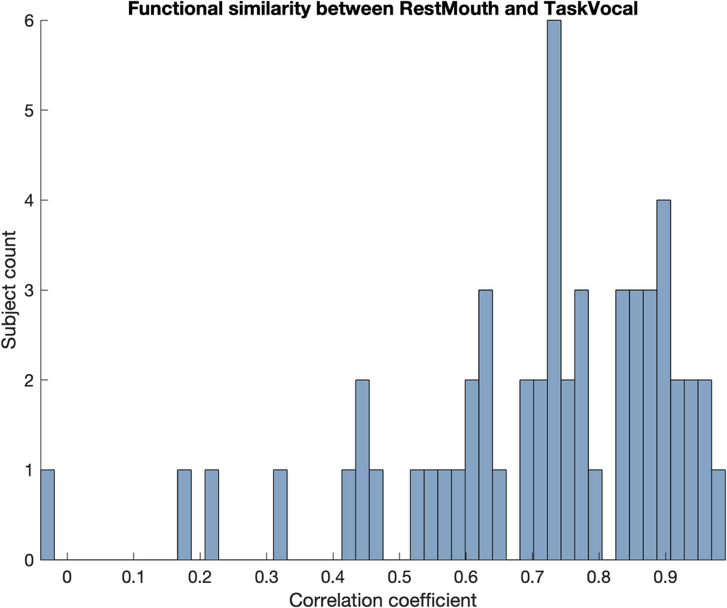


**Supplementary Figure 6: Functional similarity between task-based and resting-state networks within subjects.** There is general agreement between task-based and resting-state effector networks, as spatially illustrated in Fig. 3A in the manuscript. Here, we quantify their functional similarity by plotting the Pearson’s correlation between the average BOLD timeseries in task-based vs. resting-state networks within subjects. Most subjects demonstrated moderate (>0.6) to high (>0.8) correlation and the consistency was greater for Hand compared to Mouth region.


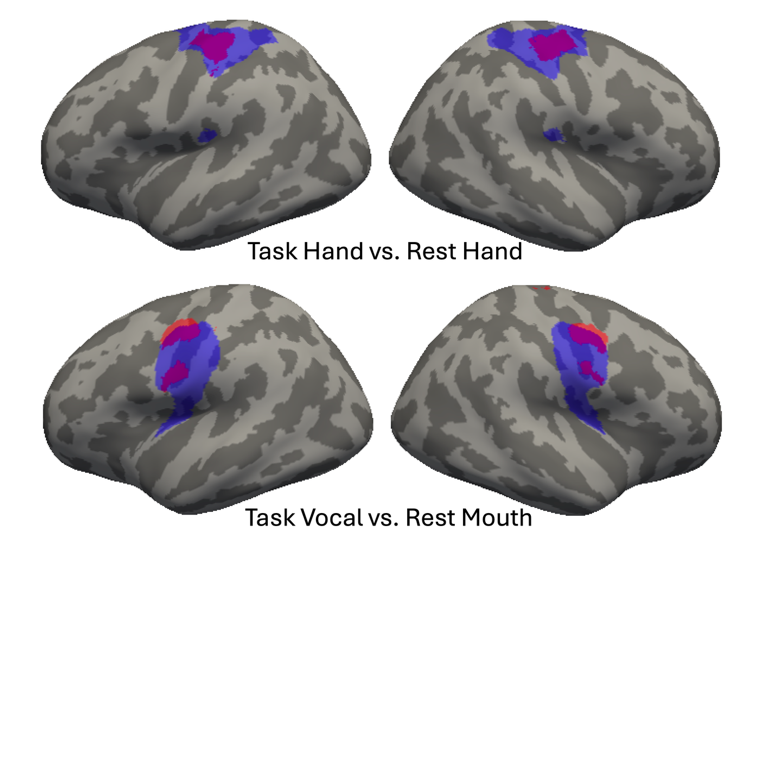


**Supplementary Figure 7: Spatial similarity between task-based (red) and resting-state (blue) networks on a group level.** The threshold was set at 80% group overlap; all colored regions represent areas where a given network was identified in at least 80% of participants whose functional ROIs passed quality control (*N*=57 for Task Hand, *N*=56 for Task Vocal, *N*=61 for resting-state parcellation). There is general agreement between task-based and resting-state effector networks, and the consistency was greater for Hand compared to Mouth region.

**Supplementary Table 1: Cortico-cortical FC (linear model 1) with task-based effector ROI**

|  | **FC** | | |
| --- | --- | --- | --- |
| *Predictors* | *Estimates* | *CI* | *P* |
| (Intercept) | 0.42 | 0.23 – 0.60 | **<0.001** |
| group [LD] | -0.07 | -0.23 – 0.09 | 0.368 |
| group [FHD] | -0.09 | -0.26 – 0.07 | 0.264 |
| ROIpair [Foot-SCAN] | 0.15 | 0.01 – 0.30 | **0.038** |
| ROIpair [TaskHand-SCAN] | 0.10 | -0.04 – 0.25 | 0.170 |
| ROIpair [TaskVocal-SCAN] | 0.29 | 0.15 – 0.44 | **<0.001** |
| age | 0.00 | -0.00 – 0.00 | 0.474 |
| sex [M] | 0.01 | -0.06 – 0.08 | 0.860 |
| symptom duration | -0.00 | -0.00 – 0.00 | 0.742 |
| Mean Relative Motion | -0.07 | -0.59 – 0.45 | 0.799 |
| group [LD] × ROIpair  [Foot-SCAN] | 0.06 | -0.14 – 0.27 | 0.536 |
| group [FHD] × ROIpair  [Foot-SCAN] | 0.12 | -0.10 – 0.34 | 0.273 |
| group [LD] × ROIpair  [TaskHand-SCAN] | 0.06 | -0.15 – 0.26 | 0.596 |
| group [FHD] × ROIpair  [TaskHand-SCAN] | 0.11 | -0.10 – 0.33 | 0.305 |
| group [LD] × ROIpair  [TaskVocal-SCAN] | 0.21 | 0.00 – 0.41 | **0.048** |
| group [FHD] × ROIpair  [TaskVocal-SCAN] | 0.26 | 0.05 – 0.48 | **0.017** |
| Observations | 244 | | |
| R^2^ / R^2^ adjusted | 0.336 / 0.292 | | |

**Supplementary Table 2: Cortico-cortical FC (linear model 1) with resting-state effector ROI**

|  | **FC** | | |
| --- | --- | --- | --- |
| *Predictors* | *Estimates* | *CI* | *p* |
| (Intercept) | 0.48 | 0.32 – 0.65 | **<0.001** |
| group [LD] | -0.03 | -0.17 – 0.10 | 0.626 |
| group [FHD] | -0.06 | -0.20 – 0.08 | 0.409 |
| ROIpair [Foot-SCAN] | 0.15 | 0.03 – 0.28 | **0.016** |
| ROIpair [RestHand-SCAN] | 0.06 | -0.07 – 0.19 | 0.352 |
| ROIpair [RestMouth-SCAN] | 0.09 | -0.04 – 0.21 | 0.170 |
| age | 0.00 | -0.00 – 0.00 | 0.999 |
| sex [M] | 0.01 | -0.05 – 0.07 | 0.711 |
| symptom duration | -0.00 | -0.00 – 0.00 | 0.152 |
| Mean Relative Motion | -0.24 | -0.69 – 0.21 | 0.301 |
| group [LD] × ROIpair  [Foot-SCAN] | 0.06 | -0.11 – 0.24 | 0.472 |
| group [FHD] × ROIpair  [Foot-SCAN] | 0.12 | -0.07 – 0.31 | 0.204 |
| group [LD] × ROIpair  [RestHand-SCAN] | 0.13 | -0.05 – 0.30 | 0.157 |
| group [FHD] × ROIpair  [RestHand-SCAN] | 0.13 | -0.05 – 0.32 | 0.165 |
| group [LD] × ROIpair  [RestMouth-SCAN] | 0.08 | -0.10 – 0.25 | 0.401 |
| group [FHD] × ROIpair  [RestMouth-SCAN] | 0.14 | -0.05 – 0.32 | 0.152 |
| Observations | 244 | | |
| R^2^ / R^2^ adjusted | 0.157 / 0.102 | | |

**Supplementary Table 3: Cortico-subcortical-cerebellar FC (linear model 2) with task-based effector ROI.**

|  | **FC** | | |
| --- | --- | --- | --- |
| *Predictors* | *Estimates* | *CI* | *p* |
| (Intercept) | 0.11 | 0.01 – 0.20 | **0.027** |
| age | -0.00 | -0.00 – 0.00 | 0.328 |
| sex [M] | 0.01 | -0.01 – 0.04 | 0.351 |
| Mean Relative Motion | -0.40 | -0.59 – -0.21 | **<0.001** |
| symptom duration | -0.00 | -0.00 – -0.00 | **0.001** |
| group [LD] | 0.03 | -0.08 – 0.14 | 0.607 |
| group [FHD] | -0.01 | -0.12 – 0.11 | 0.891 |
| ROIpair [SCAN-THa] | -0.00 | -0.11 – 0.11 | 0.999 |
| ROIpair [SCAN-Putm] | 0.11 | -0.00 – 0.21 | 0.055 |
| ROIpair [SCAN-smCB] | -0.12 | -0.23 – -0.02 | **0.024** |
| ROIpair [Foot-THp] | -0.02 | -0.13 – 0.08 | 0.673 |
| ROIpair [Foot-THa] | -0.08 | -0.19 – 0.03 | 0.141 |
| ROIpair [Foot-Putm] | -0.01 | -0.12 – 0.09 | 0.813 |
| ROIpair [Foot-smCB] | -0.09 | -0.20 – 0.01 | 0.083 |
| ROIpair [TaskHand-THp] | -0.06 | -0.16 – 0.05 | 0.309 |
| ROIpair [TaskHand-THa] | -0.17 | -0.28 – -0.07 | **0.002** |
| ROIpair [TaskHand-Putm] | -0.13 | -0.24 – -0.02 | **0.019** |
| ROIpair [TaskHand-smCB] | -0.11 | -0.22 – -0.00 | **0.049** |
| ROIpair [TaskVocal-THp] | -0.05 | -0.16 – 0.06 | 0.377 |
| ROIpair [TaskVocal-THa] | -0.07 | -0.18 – 0.03 | 0.180 |
| ROIpair [TaskVocal-Putm] | 0.05 | -0.05 – 0.16 | 0.320 |
| ROIpair [TaskVocal-smCB] | -0.17 | -0.28 – -0.06 | **0.002** |
| group [LD] × ROIpair  [SCAN-THa] | 0.01 | -0.14 – 0.16 | 0.917 |
| group [FHD] × ROIpair  [SCAN-THa] | -0.06 | -0.22 – 0.10 | 0.434 |
| group [LD] × ROIpair  [SCAN-Putm] | 0.03 | -0.12 – 0.18 | 0.729 |
| group [FHD] × ROIpair  [SCAN-Putm] | 0.04 | -0.12 – 0.20 | 0.610 |
| group [LD] × ROIpair  [SCAN-smCB] | 0.12 | -0.03 – 0.27 | 0.111 |
| group [FHD] × ROIpair  [SCAN-smCB] | 0.14 | -0.02 – 0.30 | 0.088 |
| group [LD] × ROIpair  [Foot-THp] | -0.01 | -0.16 – 0.14 | 0.886 |
| group [FHD] × ROIpair  [Foot-THp] | -0.01 | -0.17 – 0.15 | 0.918 |
| group [LD] × ROIpair  [Foot-THa] | -0.02 | -0.17 – 0.13 | 0.819 |
| group [FHD] × ROIpair  [Foot-THa] | -0.08 | -0.24 – 0.08 | 0.340 |
| group [LD] × ROIpair  [Foot-Putm] | -0.02 | -0.17 – 0.13 | 0.754 |
| group [FHD] × ROIpair  [Foot-Putm] | 0.01 | -0.15 – 0.17 | 0.924 |
| group [LD] × ROIpair  [Foot-smCB] | -0.01 | -0.16 – 0.14 | 0.913 |
| group [FHD] × ROIpair  [Foot-smCB] | 0.08 | -0.08 – 0.24 | 0.332 |
| group [LD] × ROIpair  [TaskHand-THp] | 0.01 | -0.14 – 0.16 | 0.918 |
| group [FHD] × ROIpair  [TaskHand-THp] | 0.04 | -0.12 – 0.20 | 0.618 |
| group [LD] × ROIpair  [TaskHand-THa] | -0.00 | -0.15 – 0.15 | 0.965 |
| group [FHD] × ROIpair  [TaskHand-THa] | -0.04 | -0.20 – 0.12 | 0.596 |
| group [LD] × ROIpair  [TaskHand-Putm] | 0.01 | -0.14 – 0.16 | 0.930 |
| group [FHD] × ROIpair  [TaskHand-Putm] | 0.08 | -0.08 – 0.24 | 0.326 |
| group [LD] × ROIpair  [TaskHand-smCB] | 0.05 | -0.10 – 0.20 | 0.545 |
| group [FHD] × ROIpair  [TaskHand-smCB] | 0.09 | -0.07 – 0.25 | 0.258 |
| group [LD] × ROIpair  [TaskVocal-THp] | 0.04 | -0.11 – 0.19 | 0.615 |
| group [FHD] × ROIpair  [TaskVocal-THp] | 0.04 | -0.12 – 0.20 | 0.644 |
| group [LD] × ROIpair  [TaskVocal-THa] | 0.05 | -0.10 – 0.20 | 0.518 |
| group [FHD] × ROIpair  [TaskVocal-THa] | -0.01 | -0.17 – 0.14 | 0.854 |
| group [LD] × ROIpair  [TaskVocal-Putm] | 0.02 | -0.13 – 0.17 | 0.838 |
| group [FHD] × ROIpair  [TaskVocal-Putm] | 0.07 | -0.09 – 0.23 | 0.385 |
| group [LD] × ROIpair  [TaskVocal-smCB] | 0.12 | -0.03 – 0.27 | 0.117 |
| group [FHD] × ROIpair  [TaskVocal-smCB] | 0.12 | -0.03 – 0.28 | 0.122 |
| Observations | 976 | | |
| R^2^ / R^2^ adjusted | 0.195 / 0.150 | | |

**Supplementary Table 4: Cortico-subcortical-cerebellar FC (linear model 2) with resting-state effector ROI.**

|  | **FC** | | |
| --- | --- | --- | --- |
| *Predictors* | *Estimates* | *CI* | *p* |
| (Intercept) | 0.11 | 0.01 – 0.21 | **0.028** |
| age | -0.00 | -0.00 – 0.00 | 0.362 |
| sex [M] | 0.01 | -0.02 – 0.03 | 0.626 |
| Mean Relative Motion | -0.39 | -0.58 – -0.19 | **<0.001** |
| symptom duration | -0.00 | -0.00 – -0.00 | **<0.001** |
| group [LD] | 0.03 | -0.08 – 0.14 | 0.600 |
| group [FHD] | -0.00 | -0.12 – 0.11 | 0.947 |
| ROIpair [SCAN-THa] | -0.00 | -0.11 – 0.11 | 0.999 |
| ROIpair [SCAN-Putm] | 0.11 | -0.00 – 0.21 | 0.061 |
| ROIpair [SCAN-smCB] | -0.12 | -0.23 – -0.01 | **0.027** |
| ROIpair [Foot-THp] | -0.02 | -0.13 – 0.09 | 0.680 |
| ROIpair [Foot-THa] | -0.08 | -0.19 – 0.03 | 0.150 |
| ROIpair [Foot-Putm] | -0.01 | -0.12 – 0.10 | 0.817 |
| ROIpair [Foot-smCB] | -0.09 | -0.20 – 0.02 | 0.090 |
| ROIpair [RestHand-THp] | -0.07 | -0.18 – 0.04 | 0.199 |
| ROIpair [RestHand-THa] | -0.19 | -0.30 – -0.08 | **0.001** |
| ROIpair [RestHand-Putm] | -0.12 | -0.23 – -0.01 | **0.028** |
| ROIpair [RestHand-smCB] | -0.10 | -0.21 – 0.01 | 0.066 |
| ROIpair [RestMouth-THp] | -0.01 | -0.12 – 0.10 | 0.849 |
| ROIpair [RestMouth-THa] | -0.09 | -0.20 – 0.02 | 0.108 |
| ROIpair [RestMouth-Putm] | -0.02 | -0.13 – 0.09 | 0.753 |
| ROIpair [RestMouth-smCB] | -0.17 | -0.28 – -0.06 | **0.002** |
| group [LD] × ROIpair  [SCAN-THa] | 0.01 | -0.15 – 0.16 | 0.919 |
| group [FHD] × ROIpair  [SCAN-THa] | -0.06 | -0.22 – 0.10 | 0.444 |
| group [LD] × ROIpair  [SCAN-Putm] | 0.03 | -0.13 – 0.18 | 0.735 |
| group [FHD] × ROIpair  [SCAN-Putm] | 0.04 | -0.12 – 0.20 | 0.618 |
| group [LD] × ROIpair  [SCAN-smCB] | 0.12 | -0.03 – 0.28 | 0.120 |
| group [FHD] × ROIpair  [SCAN-smCB] | 0.14 | -0.02 – 0.30 | 0.095 |
| group [LD] × ROIpair  [Foot-THp] | -0.01 | -0.16 – 0.14 | 0.888 |
| group [FHD] × ROIpair  [Foot-THp] | -0.01 | -0.17 – 0.15 | 0.920 |
| group [LD] × ROIpair  [Foot-THa] | -0.02 | -0.17 – 0.14 | 0.823 |
| group [FHD] × ROIpair  [Foot-THa] | -0.08 | -0.24 – 0.08 | 0.351 |
| group [LD] × ROIpair  [Foot-Putm] | -0.02 | -0.18 – 0.13 | 0.759 |
| group [FHD] × ROIpair  [Foot-Putm] | 0.01 | -0.15 – 0.17 | 0.926 |
| group [LD] × ROIpair  [Foot-smCB] | -0.01 | -0.16 – 0.15 | 0.915 |
| group [FHD] × ROIpair  [Foot-smCB] | 0.08 | -0.08 – 0.24 | 0.343 |
| group [LD] × ROIpair  [RestHand-THp] | 0.01 | -0.14 – 0.16 | 0.909 |
| group [FHD] × ROIpair  [RestHand-THp] | 0.03 | -0.13 – 0.19 | 0.698 |
| group [LD] × ROIpair  [RestHand-THa] | -0.03 | -0.18 – 0.12 | 0.706 |
| group [FHD] × ROIpair  [RestHand-THa] | -0.05 | -0.21 – 0.11 | 0.559 |
| group [LD] × ROIpair  [RestHand-Putm] | 0.01 | -0.14 – 0.16 | 0.907 |
| group [FHD] × ROIpair  [RestHand-Putm] | 0.06 | -0.10 – 0.23 | 0.441 |
| group [LD] × ROIpair  [RestHand-smCB] | 0.02 | -0.14 – 0.17 | 0.838 |
| group [FHD] × ROIpair  [RestHand-smCB] | 0.08 | -0.08 – 0.25 | 0.303 |
| group [LD] × ROIpair  [RestMouth-THp] | 0.02 | -0.13 – 0.18 | 0.768 |
| group [FHD] × ROIpair  [RestMouth-THp] | 0.05 | -0.11 – 0.21 | 0.530 |
| group [LD] × ROIpair  [RestMouth-THa] | 0.01 | -0.14 – 0.17 | 0.871 |
| group [FHD] × ROIpair  [RestMouth-THa] | -0.02 | -0.19 – 0.14 | 0.765 |
| group [LD] × ROIpair  [RestMouth-Putm] | -0.01 | -0.16 – 0.15 | 0.945 |
| group [FHD] × ROIpair  [RestMouth-Putm] | 0.06 | -0.10 – 0.22 | 0.480 |
| group [LD] × ROIpair  [RestMouth-smCB] | 0.08 | -0.07 – 0.24 | 0.284 |
| group [FHD] × ROIpair  [RestMouth-smCB] | 0.12 | -0.04 – 0.28 | 0.155 |
| Observations | 976 | | |
| R^2^ / R^2^ adjusted | 0.188 / 0.144 | | |

**Supplementary Table 5: Subcortical-cerebellar FC (linear model 3).**

|  | **FC** | | |
| --- | --- | --- | --- |
| *Predictors* | *Estimates* | *CI* | *p* |
| (Intercept) | 0.83 | 0.73 – 0.93 | **<0.001** |
| age | -0.00 | -0.00 – 0.00 | 0.125 |
| sex [M] | 0.02 | -0.02 – 0.05 | 0.324 |
| Mean Relative Motion | 0.53 | 0.29 – 0.78 | **<0.001** |
| symptom duration | 0.00 | 0.00 – 0.00 | **0.047** |
| group [LD] | -0.01 | -0.12 – 0.09 | 0.823 |
| group [FHD] | -0.03 | -0.14 – 0.08 | 0.626 |
| ROIpair [GPe-STN] | -0.28 | -0.39 – -0.18 | **<0.001** |
| ROIpair [GPi-STN] | -0.26 | -0.36 – -0.15 | **<0.001** |
| ROIpair [GPi-THa] | -0.52 | -0.62 – -0.41 | **<0.001** |
| ROIpair [GPi-THp] | -0.57 | -0.67 – -0.46 | **<0.001** |
| ROIpair [Putm-GPi] | -0.44 | -0.55 – -0.34 | **<0.001** |
| ROIpair [Putm-Caud] | -0.06 | -0.17 – 0.04 | 0.235 |
| ROIpair [smCB-THa] | -0.72 | -0.83 – -0.62 | **<0.001** |
| ROIpair [smCB-THp] | -0.80 | -0.90 – -0.69 | **<0.001** |
| group [LD] × ROIpair  [GPe-STN] | 0.02 | -0.12 – 0.16 | 0.782 |
| group [FHD] × ROIpair  [GPe-STN] | -0.03 | -0.18 – 0.12 | 0.717 |
| group [LD] × ROIpair  [GPi-STN] | 0.00 | -0.14 – 0.14 | 0.999 |
| group [FHD] × ROIpair  [GPi-STN] | -0.03 | -0.18 – 0.13 | 0.737 |
| group [LD] × ROIpair  [GPi-THa] | -0.12 | -0.26 – 0.03 | 0.116 |
| group [FHD] × ROIpair  [GPi-THa] | -0.03 | -0.18 – 0.12 | 0.666 |
| group [LD] × ROIpair  [GPi-THp] | -0.09 | -0.24 – 0.05 | 0.211 |
| group [FHD] × ROIpair  [GPi-THp] | -0.06 | -0.21 – 0.09 | 0.437 |
| group [LD] × ROIpair  [Putm-GPi] | -0.05 | -0.19 – 0.10 | 0.518 |
| group [FHD] × ROIpair  [Putm-GPi] | -0.01 | -0.16 – 0.14 | 0.891 |
| group [LD] × ROIpair  [Putm-Caud] | 0.01 | -0.14 – 0.15 | 0.916 |
| group [FHD] × ROIpair  [Putm-Caud] | -0.05 | -0.20 – 0.10 | 0.515 |
| group [LD] × ROIpair  [smCB-THa] | -0.04 | -0.18 – 0.10 | 0.577 |
| group [FHD] × ROIpair  [smCB-THa] | -0.03 | -0.18 – 0.12 | 0.684 |
| group [LD] × ROIpair  [smCB-THp] | 0.01 | -0.13 – 0.16 | 0.876 |
| group [FHD] × ROIpair  [smCB-THp] | -0.04 | -0.19 – 0.11 | 0.613 |
| Observations | 549 | | |
| R^2^ / R^2^ adjusted | 0.733 / 0.717 | | |

**Supplementary Table 6: SCAN-to-sensorimotor-cerebellum linear model with combined dystonia group.**

|  | **FC** | | |
| --- | --- | --- | --- |
| *Predictors* | *Estimates* | *CI* | *p* |
| (Intercept) | 0.10 | -0.15 – 0.34 | 0.426 |
| group2 [Dystonia] | 0.18 | 0.05 – 0.32 | **0.010** |
| age | -0.00 | -0.01 – 0.00 | 0.443 |
| sex [M] | 0.02 | -0.08 – 0.12 | 0.647 |
| symptom duration | -0.00 | -0.01 – 0.00 | 0.182 |
| Mean Relative Motion | -0.97 | -1.76 – -0.18 | **0.017** |
| Observations | 61 | | |
| R^2^ / R^2^ adjusted | 0.204 / 0.132 | | |
